# Notes and News

**Published:** 1886-10-09

**Authors:** 


### Notes and News.

Collected and Communicated.

The Local Government Board have refused to sanction the expenditure of £116,000 by the Metropolitan Asylums Board, for a Convalescent Hospital on the Gore Farm at Darenth. It is maintained by the opponents of the Asylums Board proposal that it can be shown that all requirements can be fully and efficiently met by the provision of temporary buildings, or huts, at a cost of something like £25,000.

A new wing is shortly to be added to the Sunderland Infirmary, in memory of the late Mr. James Hartley, at a cost of about £9,000.

Mr. Frewer stated at the last fortnightly meeting of the delegates of the Hospital Saturday Fund that the amount to hand this year was about £1,500 in excess of the amount received to the same date last year. The unfavourable weather made the street collection bad, but the workshop collections have happily more than compensated for this.

Dr. Millington's retirement from the office of Senior Physician to the Wolverhampton and Staffordshire Hospital, after an association with that institution of over thirty years, has been gracefully recognised by his many friends, who have just presented him with a silver salver and service of plate, whilst an oil painting of the Doctor goes to adorn the walls of the institution.

Someone calculates that the amount subscribed this year to the Hospital Sunday Fund represents about one ten-thousandth part of the income of the metropolis. The calculation is unquestionably ingenious, but one would like "to know, you know," how this living Babbage has arrived at the "income of the metropolis."

The Balloon Society extends its protecting wing over a multiplicity of objects, often as divergent as the poles. The recent lecture by Miss Alice Kelman, on Hospitals and Hospital Nursing, was eminently practical, and contained within a brief compass a great deal of useful information. But perhaps this lady's valuable experience might be attended with more profit if made the subject of lectures to the nursing profession, and those engaged in hospital work.

It is somewhat strange that Bath has not followed the example of other towns by devoting one Sunday in the year to the benefit of her hospitals. The Rev. M. E. Hoets, in advocating the other day the claims of the old Mineral Water Hospital, alluded to this want, a want which, it is to be hoped, will soon be supplied. Will not the good citizens of Bath come forward and help to establish a Hospital Sunday Fund? Surely Philanthropy in Bath ought not to lag behind that of smaller and more humble towns.

Dr. Collie has at last been restored to the position from which he ought never to have been ousted. The Eastern Hospitals scandal did not compromise the medical superintendent, but a scapegoat had to be found, and for nine months past Dr. Collie has been discharging his onerous duties under a burden of suspense. Even now his reinstatement has not been confirmed by anything like the unanimous voice of the Metropolitan Asylums Board. The Doctor's case has been a peculiarly hard one, and he has had to pay dearly for his indiscretion in not bringing the abuses connected with the lay management under the notice of the public and the Board.

The Governors of the London Hospital could have come to no wiser decision than they have done in appointing Dr. Stephen Mackenzie one of the physicians in the room of Sir Andrew Clark, who has retired by effluxion of time. Sir Andrew has held the appointment of physician to the London Hospital for twenty years, having filled the post of assistant physician for twelve years prior to 1866. Dr. Mackenzie, who has hitherto had the charge of the out-patients, was appointed assistant physician in 1874, and physician in 1881. As Mr. Carr Gomm pointed out to the Governors, the duties which the out-patients' department at the London Hospital entails on the medical officers are of no slight kind.

Our musical readers who patronise Messrs. Broadwood's manufactory must have seen a notice-board on a house in Great Pulteney

Street bearing the following inscription :—"London Rheumatic Hospital for the Treatment of Rheumatism, Rheumatic Gout, Neuralgia, Sciatica, and Nervous and Chronic Diseases generally. Hours of attendance, etc., etc." We wonder who constitute the medical staff of the London Rheumatic Hospital!

Dr. Ellis, of the Newcastle Throat and Ear Hospital, has just been presented with an elegant silver claret jug and salver by the students who have attended the recent course of lectures which he has been giving at this institution.

Arrangements are now well advanced for the grand afternoon concert and monster gala entertainment which it has been arranged to hold next Saturday in the North London Colosseum at Dalston Junction, in aid of the funds of the Metropolitan Free Hospital in the Kingsland Road. The recent demonstration in aid of this institution realised £139.

The Royal Free Hospital was recently, thanks to the kindness of Mr. Scott, the recipient of the welcome present of a quarter of a hundred bunches of grapes grown upon a very old vine in St. George's Gardens. It is to be hoped that Mr. Scott's practical kindness will find numerous imitators.

A correspondent of last week's *Bat*, who rejoices in anonymity, narrates his "painful experiences" of a three weeks' sojourn at Charing Cross Hospital, whither he was taken after an accident by the police. The story is a very remarkable one throughout, and to our mind certainly does not bear the impress of truth. The state of things he describes at Charing Cross Hospital would have been a disgrace to such an institution even fifty years ago. The writer frankly admits that he has "never given a cent to a hospital, and never will." These "painful experiences," just revealed to the readers of the *Bat*, *were suffered five years ago!*

A few weeks ago, at one of the largest of the London hospitals, one of the dressers was a little beyond his usual time in entering the ward in the morning. He asked the nurse to bring him the usual appliances for his work, and on her not doing so he went to the sister's room. The sister, with all the dignity she could command, and her powers in this direction are by no means contemptible, pointed out that he was very late, and she could not allow the order of the ward to be upset because he was not at his work at the usual hour. We understand, however, although the dressing of the patients was not proceeded with, that there is not to be another nursing scandal in consequence.

A lady recently wrote to a provincial Home for Nurses for a trained nurse who was capable of attending to a baby under one year old. The payment was to be one guinea per week, with travelling expenses, and all found. This nurse remained three weeks in charge of the baby, a remarkably healthy child, which, under her care (?), owing to improper feeding, was brought to death's door. She left empty laudanum bottles behind her. It was proved that when out with the children she used to lie down on a seat and go to sleep, and that she had no certificate as a trained nurse. She was, however, supplied by a provincial nursing institution, and for the credit of these establishments it ought not to be possible for any gentleman's family for the future to be supplied with a similar specimen of the kind of nurses they send out to nurse private families. None but certificated nurses of high character ought to be engaged by nursing institutions; and it would be well if all were to follow the example of the Norwich Home, which selects young women to be trained for its work at one of the large general hospitals.

The want of funds is still a marked feature in connection with the hospitals. Sir Sydney Waterlow has pointed out that the hospitals of London have some two thousand beds empty owing to the absence of wherewithal to maintain them fully occupied. To take one example out of many, it is stated that two of the thirteen wards of the Royal Westminster Ophthalmic Hospital are closed at the present time from want of funds.

"Noble's Isle of Man Hospital" has a sonorous ring about it, which attracts attention. It is the name of a new hospital and dispensary for the Isle of Man in course of erection at Douglas, on a site given by Mr. and Mrs. H. B. Noble, who have also contributed £5,000 in money. The new hospital will cost £4,038. Plans have been prepared by Messrs. Bleakley and Cubbon, architects, of Birkenhead.

Enterprise is the soul of business, and the Dean and Faculty of St. Mary's Hospital seem alive to the fact. Twelve months ago they determined to meet the views of the parents of the students by opening a residential college, although the step entailed a heavy expenditure on the part of the staff. They placed the college under the supervision of a warden, who, with the lady superintendent, resides in the building.

Such a spirited policy deserves success, and it is satisfactory to note from this year's prospectus that the medical school buildings, though enlarged only three years ago, are now found to be quite inadequate to the present demands upon them.

At Middlesex the staff and committee are co-operating, in the hope of soon being able to follow the example set by St. Mary's. At the dinner of the staff and the present and past students of Middlesex Hospital, held at the Holborn Restaurant on Tuesday, it was stated that the funds had come in freely, and that the first stone of the new college buildings would be laid without loss of time. There can be no question that the more the directors of the medical schools look after the interests of the students, the more students will parents provide for them to look after.

West Ham, situated in the poorest portion of the East-end of London, is showing commendable public spirit. The working men have determined to subscribe funds for the purpose of providing a hospital for this overcrowded borough. When H.R.H. the Duke of Cambridge and the Marquis of Salisbury visited it on behalf of all the hospitals in July last, they met with an enthusiastic reception from a crowded audience.

On Saturday West Ham was *en fête* on its own account. The Lord Mayor and Lady Mayoress attended a monster gathering of children, organised by the South Essex Auxiliary Sunday School Union. The object of the demonstration was to assist in the establishment of a hospital for the borough, and Sunday-school children of every denomination took part in the proceedings. Between five and six thousand children are estimated to have been present, in addition to some ten thousand spectators, and in the result upwards of £40 was obtained for the hospital fund.

The Mayor of Bideford, Mr. Alexander G. Duncan, has set a good example to provincial mayors throughout the country. The Bideford Dispensary and Infirmary is making an effort to raise £750 to complete the payment for the new infirmary buildings, now in course of erection. The mayor writes: "On thinking over how I could, as mayor, aid the committee in its efforts to obtain the balance required, I thought I could best do so by offering to contribute ten per cent. upon all subscriptions from this date." A vigorous effort was required to obtain the £750, and the mayor's generosity and example cannot fail to secure the desired result.

